# Supplementary material for: Polymorphism, Expression, and Structure Analysis of a Key Gene ARNT in Sheep (Ovis aries)
Source: Biology (Basel). 2022 Dec 10;11(12):1795. doi: 10.3390/biology11121795 (PMC9774921; doi:10.3390/biology11121795)
Supplement: Supplementary file 1 [file biology-11-01795-s001.zip › biology-2054064-supplementary Table S2.pdf]

**Table S2**. R code for phenotype data correction.

```
########## Phenotype Correction ############
#Session-SWD-choose dirctory
data=read.csv("5wtc.csv",header = T)#
dim(data) #
str(data) #
head(data) #
for( i in 1:5) data[,i] = as.factor(data[,i]) #
data$date5=as.numeric(as.character(data$date5)) #
str(data)
summary(data) #

a1=glm(wtc~type+year+quarter+date5,data=data) #
anova(a1,test="F")###
a1=glm(wtc~quarter+date5,data=data) #
bw_adjust=matrix(a1$residuals,ncol=1)#
head(bw_adjust) #
#
colnames(bw_adjust)=c("phe_wtc")
head(bw_adjust)
dim(bw_adjust)
#
bw_adjust=as.data.frame(cbind(data[,1:8],bw_adjust))
head(bw_adjust)
write.csv(bw_adjust,"5wtcj.csv",row.names = F) #
```
